# Emergence of Multiple Superconducting Phases in (NH_3_)_y_M_x_FeSe (M: Na and Li)

**DOI:** 10.1038/srep12774

**Published:** 2015-08-04

**Authors:** Lu Zheng, Xiao Miao, Yusuke Sakai, Masanari Izumi, Hidenori Goto, Saki Nishiyama, Eri Uesugi, Yuichi Kasahara, Yoshihiro Iwasa, Yoshihiro Kubozono

**Affiliations:** 1 Research Laboratory for Surface Science, Okayama University, Okayama 700-8530, Japan; 2 Department of Applied physics, The University of Tokyo, Tokyo 113-8564, Japan; 3 Research Centre of New Functional Materials for Energy Production, Storage and Transport, Okayama University, Okayama 700-8530, Japan

## Abstract

We previously discovered multiple superconducting phases in the ammoniated Na-doped FeSe material, (NH_3_)_y_Na_x_FeSe. To clarify the origin of the multiple superconducting phases, the variation of *T*_c_ was fully investigated as a function of x in (NH_3_)_y_Na_x_FeSe. The 32 K superconducting phase is mainly produced in the low-x region below 0.4, while only a single phase is observed at x  =  1.1, with *T*_c_ =  45 K, showing that the *T*_c_ depends significantly on x, but it changes discontinuously with x. The crystal structure of (NH_3_)_y_Na_x_FeSe does not change as x increases up to 1.1, *i.e.*, the space group of *I*4/mmm. The lattice constants, *a* and *c*, of the low-*T*_c_ phase (*T*_c_ = 32.5 K) are 3.9120(9) and 14.145(8) Å, respectively, while *a* = 3.8266(7) Å and *c* = 17.565(9) Å for the high-*T*_c_ phase (~46 K). The *c* increases in the high *T*_c_ phase, implying that the *T*_c_ is directly related to *c*. In (NH_3_)_y_Li_x_FeSe material, the *T*_c_ varies continuously within the range of 39 to 44 K with changing x. Thus, the behavior of *T*_c_ is different from that of (NH_3_)_y_Na_x_FeSe. The difference may be due to the difference in the sites that the Na and Li occupy.

Any strategy for increasing the superconducting transition temperature (*T*_c_) in metal-doped FeSe is a most exciting research subject, because the *T*_c_ can be effectively increased in metal-doped FeSe using a variety of methods such as ammoniation^1^,^2^,^3^,^4^,^5^, pressure application^6^ and solvent intercalation^7^; FeSe corresponds to PbO-type FeSe, *i.e.*, β-FeSe. The highest *T*_c_ reported in bulk superconductors of metal-doped FeSe is ~50 K, as recorded in Tl_0.6_Rb_0.4_Fe_1.67_Se_2_ and K_0.8_Fe_1_._7_Se_2_^6^ under high pressure; through this paper, the chemical representation of (NH_3_)_y_M_x_FeSe is used instead of (NH_3_)_y_M_x’_Fe_2_Se_2_. Our representation refers to the primitive basis of the crystal. Therefore, x = x’/2. At ambient pressure, the highest *T*_c_ in metal-doped FeSe materials is 46 K for (NH_3_)_y_Na_0.5_FeSe^1^. In metal-doped FeSe, the *T*_c_ increases with an increase in the lattice constant *c*, or the plane spacing between FeSe layers. The insertion of NH_3_ molecules or ammoniated metal coordinates between the FeSe layers can expand the *c*. The ammoniation and metal intercalation in FeSe (or the formation of (NH_3_)_y_M_x_FeSe) can be simultaneously achieved by dissolving metal and FeSe in liquid NH_3_ at low temperature. Recently, we succeeded in synthesizing (NH_3_)_y_M_x_FeSe_0.5_Te_0.5_ using the liquid NH_3_ technique^8^, and found that the *T*_c_ of (NH_3_)_y_Na_x_FeSe_0.5_Te_0.5_ is lower than that of the corresponding FeSe materials. Thus, the liquid NH_3_ technique is applicable to the intercalation of metal atoms into various FeSe_1-z_Te_z_ (0.0 ≤ z ≤ 1.0). The chemical doping of metal atoms and NH_3_ or ammoniated metal coordinates provides the advantage of expanding the two-dimensional (2D) layer spacing, which may contribute to an improved *T*_c_.

Recently, the presence of multiple superconducting phases has been clarified in (NH_3_)_y_K_x_FeSe^4^ and (NH_3_)_y_Na_x_FeSe^8^. In (NH_3_)_y_K_x_FeSe, a high-*T*_c_ phase (*T*_c _= 44 K) was discovered in addition to the low-*T*_c_ phase (*T*_c_ = 30 K)^4^. The x-dependence of *T*_c_ was found to involve a discontinuous change in *T*_c_; the high-*T*_c_ phase was observed with low x values, and the low-*T*_c_ phase found with high x values. The *T*_c_ was correlated with *c, i.e.*, a smaller *c* resulted in a lower *T*_c_. Discovering the presence of multiple phases is significant because of the possibility that a phase with even higher *T*_c_ might be found, even in metal-doped FeSe compositions previously examined. This makes the search for multiple superconducting phases in other FeSe materials important and exciting.

As described above, we recently discovered a new superconducting phase (low-*T*_c_ phase: *T*_c_ = 31.5 K^8^) in addition to the high-*T*_c_ phase (*T*_c_ = 46 K^1^) reported previously in (NH_3_)_y_Na_x_FeSe. However, a systematic study on the origin of the different superconducting phases has not yet been performed, although a difference in *c* was found for the two phases. Also, the relationship between *T*_c_ and x has not yet been investigated in (NH_3_)_y_Na_x_FeSe. Here we systematically study the variation of *T*_c_ as a function of x in (NH_3_)_y_Na_x_FeSe. Furthermore, the lattice constants were determined for the superconducting phases realized at different x’s to clarify the correlation between *T*_c_ and lattice constants. The variation of *T*_c_ in (NH_3_)_y_Li_x_FeSe was also investigated as a function of x. Some interesting points to be clarified are as follows: (1) the presence of multiple superconducting phases, (2) the discontinuous or continuous variation of *T*_c_, and (3) the origin of the variation of *T*_c_. In this study, the superconducting phases were identified in the (NH_3_)_y_Na_x_FeSe and (NH_3_)_y_Li_x_FeSe samples, so that the answers were provided for the above important points.

## Results

### Multiple superconducting phases in (NH_3_)_y_Na_x_FeSe

**Figure 1:**
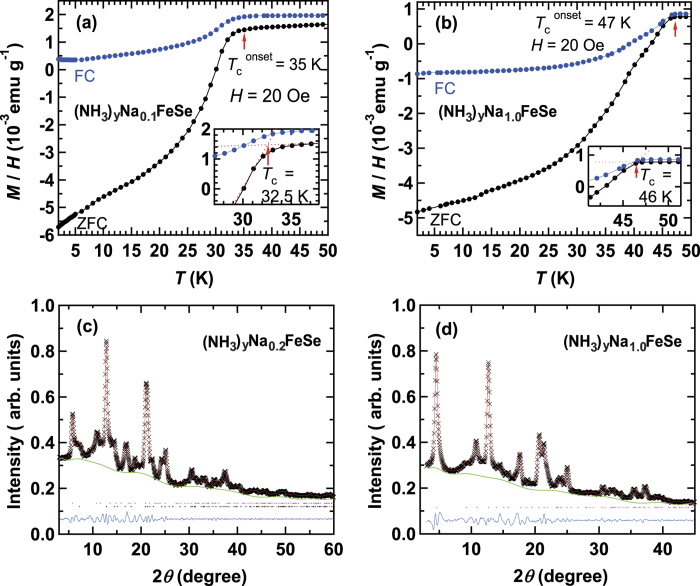
*M* / *H vs. T* plots of (**a**) (NH_3_)_y_Na_0.1_FeSe (low-*T*_c_ phase) and(**b**) (NH_3_)_y_Na_1.0_FeSe (high-*T*_c_ phase) in ZFC and FC modes (*H* = 20 Oe). Insets in (**a**) and (**b**) show the method used to determine *T*_c_. XRD patterns of (**c**) (NH_3_)_y_Na_0.2_FeSe (low-*T*_c_ phase) and (d) (NH_3_)_y_Na_1.0_FeSe (high-*T*_c_ phase); ‘x’ marks correspond to the experimental XRD patterns. Red and green lines refer to calculated patterns (LeBail fitting) and background, respectively. Ticks refer to the peak positions predicted. In (**c**), two phases ((NH_3_)_y_Na_0.2_FeSe and β-FeSe) are used in LeBail fitting, while in (**d**), a single phase ((NH_3_)_y_Na_1.0_FeSe) is used.

Figure 1(a) show the *M* / *H* curves of typical low-*T*_c_ and high-*T*_c_ phases of (NH_3_)_y_Na_x_FeSe, respectively. In the former sample nominal x = 0.1, and in the latter nominal x = 1.0. These samples each contain a ‘single phase’, either low-*T*_c_ or high-*T*_c_; actually a trace of non-doped β-FeSe (*T*_c_ = 8 K) is found in Fig. 1(a). The *T*_c_^onset^ and *T*_c_ were determined to be 35 and 32.5 K from *M* / *H* curves at ZFC for the low-*T*_c_ phase (Fig. 1(a)), while the *T*_c_^onset^ and *T*_c_ were 47 and 46 K at ZFC for the high-*T*_c_ phase (Fig. 1(b)). How the *T*_c_ was determined is shown in the insets of Fig. 1(a). The shielding fractions at 10 K were evaluated to be 30.5% for the low-*T*_c_ phase and 26% for the high *T*_c_ phase.

Figure 1(c) show the XRD patterns of the low-*T*_c_ and high-*T*_c_ phases of (NH_3_)_y_Na_x_FeSe, respectively. The *T*_c_^onset^ and *T*_c_ for the former sample (nominal x = 0.2) were determined to be 35 and 32.5 K, respectively, from the *M* / *H* curve (not shown) in ZFC mode, and the shielding fraction at 10 K was 18%. No high-*T*_c_ phase was observed in this sample, but non-doped β-FeSe was included in the sample. The latter sample (nominal x = 1.0) is the same as that providing the *M* / *H* curve shown in Fig. 1(b); no low-*T*_c_ phase was observed in the *M* / *H vs. T* curve of this sample.

The *a* and *c* were determined to be 3.9120(9) Å and 14.145(8) Å, respectively, for the low-*T*_c_ phase (Fig. 1(c)), while the *a* and *c* were 3.8266(7) Å and 17.565(9) Å respectively, for the high-*T*_c_ phase (Fig. 1(d)). The values of *a* and *c* were determined using LeBail fitting under the space group of *I*4/mmm, suggesting no change of crystal structure between the low-*T*_c_ and high-*T*_c_ phases. The *a* and *c* values for both the phases were close to those reported previously by our group (*a *= 3.891(2) Å and *c* = 14.269(4) Å for the low-*T*_c_ phase; *a* = 3.8275(6) Å and *c* = 17.579(5) Å, for the high-*T*_c_ phase)^9^. Furthermore, the *a* and *c* for the high-*T*_c_ phase determined in this study are also consistent with those reported by Ying *et al.* (*a* = 3.7846(4) Å and *c* = 17.432(1) Å)^1^. From the lattice constants, it has been concluded that a larger *c* produces a higher *T*_c_. This conclusion is supported by the relation between *T*_c_ and *c* found in (NH_3_)_y_M_x_FeSe materials with various M^5^.

### X dependence of superconductivity in (NH_3_)_y_Na_x_FeSe

To clarify the exact stoichiometry of (NH_3_)_y_Na_x_FeSe samples prepared in this study, the energy dispersive X-ray (EDX) spectroscopy was measured. To confirm the accuracy and precision of x determined by EDX, the x was determined for a reference sample, NaCl (purity = 99.0%). The EDX of NaCl showed Na_1.0(1)_Cl, which means that the EDX can provide the x value with high accuracy and precision. The typical EDX spectra of the samples containing low-*T*_c_ and high-*T*_c_ phases are shown in Figures S1 and S2 of Supplementary Information. The exact stoichiometric composition of the (NH_3_)_y_Na_0.1_FeSe sample (nominal composition of Na = 0.1) with *T*_c_ = 33.6 K (low-*T*_c_ phase) was expressed as ‘(NH_3_)_y_Na_0.17(3)_FeSe’, while the exact stoichiometric composition of the (NH_3_)_y_Na_1.0_FeSe sample (nominal composition of Na = 1.0) with *T*_c _= 45.1 K (high-*T*_c_ phase) was ‘(NH_3_)_y_Na_1.1(1)_FeSe’. These results show the consistency between the nominal and actual stoichiometry. However, the stoichiometric compositions of some samples determined from EDX spectra deviated within 0.3 from the nominal compositions, suggesting the difficulty in making the target material using liquid reaction method. Therefore, we use the x values determined from the EDX spectroscopy for x dependence of *T*_c_ and *c* in (NH_3_)_y_Na_x_FeSe samples. Notably, the exact amounts of NH_3_ contained in the samples could not be determined from EDX. Furthermore, we can comment on Fe vacancy. The EDX provided the chemical composition of Fe as 0.80(5) for (NH_3_)_y_Na_0.17(3)_FeSe, *i.e.*, it can be expressed as ‘(NH_3_)_y_Na_0.17(3)_Fe_0.80(5)_Se’ if considering the Fe vacancy. The stoichiometry of Fe was 0.70–0.84 in all samples, suggesting that ~75% of 4*d* site is occupied by Fe atom. Through this paper, we do not show the exact stoichiometry of Fe for all samples because it does not change as a function of x, *i.e.*, it does not relate to the change of lattice parameters and *T*_c_.

**Figure 2:**
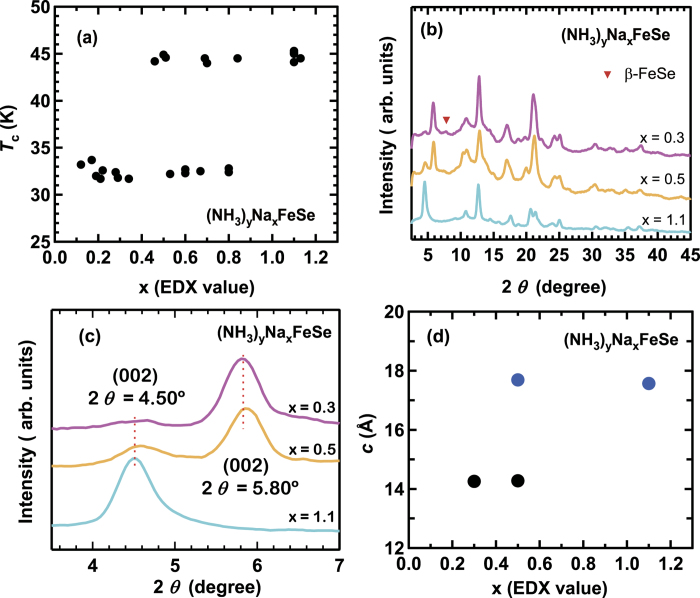
(**a**) *T*_c_ *vs*. x plots for (NH_3_)_y_Na_x_FeSe. x is determined from EDX spectra of the samples. (**b**) XRD patterns of (NH_3_)_y_Na_x_FeSe at x = 0.3, 0.5 and 1.1. (**c**) XRD peaks ascribable to 002 reflection of (NH_3_)_y_Na_x_FeSe at x = 0.3, 0.5 and 1.1. (**d**) *c* vs. x plots in (NH_3_)_y_Na_x_FeSe. Solid black and blue circles refer to low-*T*_c_ and high-*T*_c_ phases, respectively.

The *T*_c_’s of all (NH_3_)_y_Na_x_FeSe samples prepared in this study are plotted as a function of x, as shown in Fig. 2(a); the x is determined from EDX spectroscopy. In the low-x region below 0.4, the low-*T*_c_ phase (*T*_c_ = 32 K) predominates in the samples, and all the samples substantially contain either the low-*T*_c_ (*T*_c_ = 32 K) or high-*T*_c_ (*T*_c_ 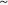 45 K) phase, *i.e.*, a predominant phase exists in each sample. The *T*_c_ of the low-*T*_c_ phase does not change throughout the entire x region. As seen from Fig. 2(a), the high-*T*_c_ phase tends to be produced in the high-x region. The *T*_c_ of the high-*T*_c_ phase does not change with an increase in x, suggesting that (NH_3_)_y_Na_x_FeSe does not show solid solution-like behavior (continuous change of *T*_c_), but discontinuous superconducting phases. Only a phase exhibiting high *T*_c_ (~45 K) is produced at x = 1.1 in (NH_3_)_y_Na_x_FeSe. At x = 0.5–0.9, either low-*T*_c_ or high-*T*_c_ phase is observed. These results show that the higher *T*_c_ is realized in the samples with higher x values. In other words, the *T*_c_ can be controlled by changing the proportion of Na (or x) in (NH_3_)_y_Na_x_FeSe. Here we briefly comment the amount of NH_3_. In this study, the NH_3_ amount was detected for only the low-*T*_c_ phase by the mass difference before and after the ammoniation. The y value of the low-*T*_c_ phase was scattered in 0.2–0.6. Nevertheless, the presence of NH_3_ was evidenced for the low-*T*_c_ phase, which is fully discussed in the **Discussion** section.

The XRD patterns of (NH_3_)_y_Na_x_FeSe at the x values of 0.3, 0.5 and 1.1 are shown in Fig. 2(b); the x values refer to those determined from EDX. A similar XRD pattern is observed in all x values, *i.e.*, the same crystal structure (*I*4/mmm). The expanded XRD patterns are shown in Fig. 2(c), exhibiting a pronounced 002 peak at 2*θ* = 5.80*°* and a very weak 002 peak at 2*θ* = 4.50° for x = 0.3 and only a peak at 2*θ* = 4.50° for x = 1.1. This means that *c* is greater when x = 1.1 than the main phase (low-*T*_c_ phase) at x = 0.3. At x = 0.5, two peaks (intense and weak peaks) are observed, at 2*θ* = 5.80° and 2*θ* = 4.50°, respectively, suggesting that two phases coexist in one sample in this x region but the high-*T*_c_ phase’s peak is small. Thus, the XRD peak discontinuously shifts from 2*θ* = 5.80° to 2*θ* = 4.50°, which implies that only two structural phases exist, even when the x value changes. In other words, a solid-solution like structure does not appear in the (NH_3_)_y_Na_x_FeSe material. The x dependence of *c* determined by a LeBail fitting for the XRD patterns is shown in Fig. 2(d), which provides a constant *c* in the low *T*_c_-phase for x = 0.3 and 0.5 and a constant *c* in the high-*T*_c_ phase for x = 0.5 and 1.1. The *a* is constant in the whole x values, indicating that the observed variation in *T*_c_ is not related to *a,* as seen from Fig. S3 of Supplementary Information.

### X dependence of superconductivity in (NH_3_)_y_Li_x_FeSe

**Figure 3:**
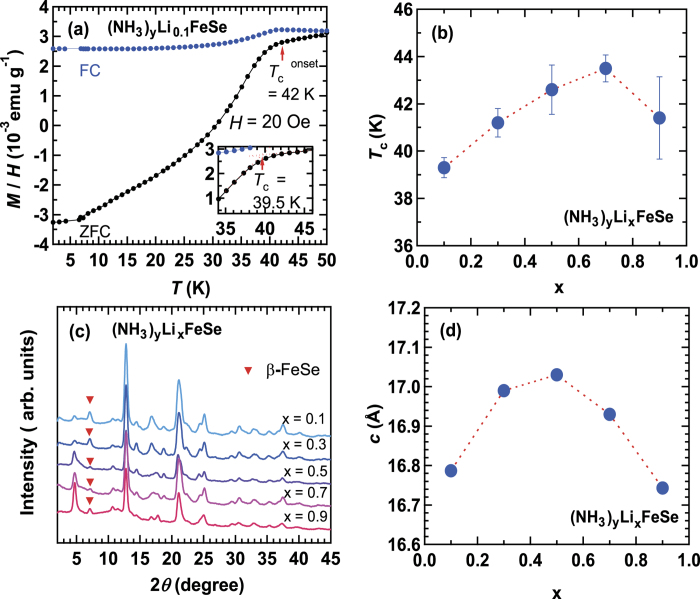
(**a**) *M* / *H vs. T* plots of (NH_3_)_y_Li_0.1_FeSe in ZFC and FC modes (*H* = 20 Oe). Inset in (a) shows the method used to determine *T*_c_. (**b**) *T*_c_ *vs*. x plot in (NH_3_)_y_Li_x_FeSe. (**c**) XRD patterns of (NH_3_)_y_Li_x_FeSe at x = 0.1 to 0.9. (**d**) *c* vs. x plots in (NH_3_)_y_Li_x_FeSe; x is nominal value.

Figure 3(a) shows the *M* / *H* curves in ZFC and FC modes for (NH_3_)_y_Li_x_FeSe at _x_ = 0.1. Notably, the x value is nominal because EDX cannot provide the exact stoichiometric composition of Li; the detection of Li peak was impossible in our EDX spectrometer because of the confined energy range. The *T*_c_^onset^ and *T*_c_ were determined to be 42 and 39.5 K from *M* / *H* curve at ZFC. The shielding fraction was evaluated to be 25.5% at 10 K. The *M* / *H vs. T* curve shows that this sample contains only a single superconducting phase. We made the (NH_3_)_y_Li_x_FeSe samples (x = 0.1 to 0.9), and when their *M* / *H* curves were measured, they showed only a single superconducting phase. The average *T*_c_, <*T*_c_>, is plotted with the estimated standard deviation (esd) as a function of x in Fig. 3(b); the <*T*_c_> was evaluated from three samples. The <*T*_c_> *vs.* x plot shows a continuous variation. It slowly increases with increasing x up to 0.7; <*T*_c_> = 43.5 K, and decreases slightly from 0.7. The maximum *T*_c_ ( = 44 K) is observed at x = 0.7. However, the variation is at most 5 K. The dome-like behavior in *T*_c_ *vs.* x plot shown in Fig. 3(b) was not observed in (NH_3_)_y_Na_x_FeSe. Thus, it has been concluded that the behavior of *T*_c_ versus x in (NH_3_)_y_M_x_FeSe is completely different depending on whether M = Na or Li. Although the nominal x was used in the plot of (NH_3_)_y_Li_x_FeSe, this conclusion should be reliable. The dome-like behavior of *T*_c_ – x (Fig. 3b) must be validated by the *T*_c_ – x plot using actual x value which may be determined by neutron diffraction.

Figure 3(c) shows the XRD patterns of (NH_3_)_y_Li_x_FeSe (x = 0.1 to 0.9); x is the nominal values. The XRD patterns of (NH_3_)_y_Li_x_FeSe are the same in all x regions. The XRD patterns could be reproduced with two phases of (NH_3_)_y_Li_x_FeSe and small amounts of β-FeSe. The *c* value, determined using LeBail fitting, gradually increases with increasing x up to 0.5, and decreases monotonically, as seen from Fig. 3(d); the maximum *c* ( = 17.03(2) Å) is observed at x = 0.5; the typical LeBail fitting for the XRD pattern is shown in Figure S4 of Supplementary Information. The results show clearly that *T*_c_ is correlated with *c*. Compared with *c* value, *a* value is almost constant (see Figure S5 of Supplementary Information). As in (NH_3_)_y_Na_x_FeSe, the larger *c* provides the higher *T*_c_ in (NH_3_)_y_Li_x_FeSe, but the *c* changes continuously in (NH_3_)_y_Li_x_FeSe, in contrast to the *c vs.* x plot in (NH_3_)_y_Na_x_FeSe. Thus, solid-solution like behavior is observed in both *T*_c_ and *c* in (NH_3_)_y_Li_x_FeSe.

## Discussion

**Figure 4:**
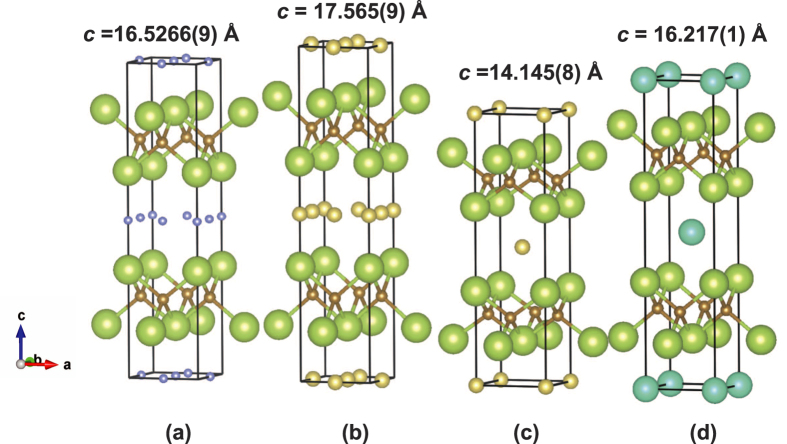
Crystal structures determined for (**a**) (NH_3_)_y_Li_x_FeSe (ref. 2) and (d) (NH_3_)_y_Cs_x_FeSe (ref. 5), and the structure suggested for (**b**) high-*T*_c_ phase and (**c**) low-*T*_c_ phase of (NH_3_)_y_Na_x_FeSe. Green, brown, blue, yellow, and dark green balls refer to Se, Fe, Li, Na and Cs, respectively. Ball sizes reflect relative ionic radii. N and H are not shown. All schematic structures are drawn by ourselves based on the crystal structures determined for (NH_3_)_y_Li_x_FeSe (ref. 2) and (NH_3_)_y_Cs_x_FeSe (ref. 5), and those of high-*T*_c_ and low-*T*_c_ phases of (NH_3_)_y_Na_x_FeSe are drawn by ourselves using atomic coordinates of (NH_3_)_y_Li_x_FeSe (ref. 2) and (NH_3_)_y_Cs_x_FeSe (ref. 5), respectively, and their lattice constants determined in this study.

Here we must consider why *c* is different in the low-*T*_c_ and high-*T*_c_ phases. As shown for (NH_3_)_y_Li_x_FeSe, with a *T*_c_ as high as 43 K, the Li atoms occupy an off-center position in the *I*4/mmm lattice (Fig. 4(a))^2^. Specifically, the Li atoms occupy the 4*c* site (0,1/2,0) and 2*b* site (0,0,1/2). The *T*_c_ (= 43 K)^2^ and the *c* ( = 16.5266(9) Å)^2^ of (NH_3_)_y_Li_x_FeSe are close to those of the high-*T*_c_ phase (*T*_c_ = 46 K and *c* = 17.565(9) Å) of (NH_3_)_y_Na_x_FeSe. The difference in *c*, Δ*c*, between (NH_3_)_y_Li_x_FeSe and the high-*T*_c_ phase of (NH_3_)_y_Na_x_FeSe may be due to the different ionic radii of Li and Na and consequent differences in local structure around M. If this is the case, it is tempting to conclude that the structure of the high-*T*_c_ phase in (NH_3_)_y_Na_x_FeSe may be the same as that of (NH_3_)_y_Li_x_FeSe (or off-center structure, Fig. 4(a)). The suggested structure of the high-*T*_c_ phase in (NH_3_)_y_Na_x_FeSe is shown in Fig. 4(b).

On the other hand, the *T*_c_ ( = 32.5 K) of the low-*T*_c_ phase in (NH_3_)_y_Na_x_FeSe may be similar to the *T*_c_ (= 31 K) of (NH_3_)_y_Cs_0.4_FeSe, in which the Cs occupies the 2*a* site (0,0,0) (or on-center position) (Fig. 4(d))^5^; our recent Rietveld refinement for XRD of (NH_3_)_y_Cs_0.4_FeSe showed that the Cs atom occupied 2*a* and N atom in NH_3_ occupied 4*c* site. Although the *c* ( = 14.145(8) Å) of the low-*T*_c_ phase is much smaller than that ( = 16.217(1) Å)^5^ of (NH_3_)_y_Cs_0.4_FeSe, the difference may be due to the different ionic radius and molecular orientation of NH_3_. Consequently, we suggest that the site occupied by the Na atom may be different between the low-*T*_c_ and high-*T*_c_ phases, *i.e.*, the former involves the on-center positon (Fig. 4(c)), while the latter the off-center position (Fig. 4(b)). This change should be reasonable because of the limited number of sites allowed for Na. Namely, the maximum x allowed for Na in the on-center structure is 0.5, while that in the off-center structure is 1.5. Consequently, when increasing x, (NH_3_)_y_Na_x_FeSe must take the off-center structure. This change probably leads to the different *c*’s that provide the different *T*_c_’s.

Very recently, Guo *et al.* showed the presence of non-ammoniated (NH_3_-free) Na_x’_Fe_2_Se_2_ (*T*_c_ = 37 K), NH_3_-poor Na_x’_Fe_2_Se_2_ (*T*_c_ = 45 K) and NH_3_-rich Na_x’_Fe_2_Se_2_ (*T*_c_ = 42 K)^9^, and that Na in NH_3_-free Na_x’_Fe_2_Se_2_ occupies the on-center position (called as ThCr_2_Si_2_ structure in Ref. 9). Although they did not report the x dependence of *T*_c_ and *c*, it was suggested in their paper that the Coulomb repulsion of Na-Na is an origin for structural destabilization in (NH_3_)_y_M_x_FeSe crystals. We can easily predict that the off-center structure would be more destabilized because of smaller Na-Na distance (nearest Na-Na distance = *a* / 2) than that (nearest Na-Na distance = *a*) of the on-center structure. Nevertheless, because of the limited number of sites for Na, the on-center structure should change to the off-center structure. We suggest that the Coulomb repulsion may be reduced in the off-center structure if the Na atoms select the positions of the 4*c* and 2*b* sites to avoid approaching each other, which would realize the off-center structure in spite of the energetic disadvantage. However, this study still leaves the question open.

Table 1 lists the structural parameters and *T*_c_ for the superconducting phases found in this study together with those reported by Guo *et al.*^9^ As seen from Table 1, the *c* ( = 14.257(7) Å) of the low-*T*_c_ phase of (NH_3_)_y_Na_0.28(3)_FeSe prepared in this study is larger by ~0.6 Å than that ( = 13.6678(4) Å) of the NH_3_-free Na_x’_Fe_2_Se_2_^9^ (_x’_ = _2_x = 0.65), which suggests that the low-*T*_c_ phase contains NH_3_ or coordinates between FeSe layers; our data listed in Table 1 correspond to those shown in Fig. 2(d). As described in the **Results** section, the value of y in (NH_3_)_y_Na_x_FeSe was suggested to be 0.2–0.6 for the samples containing the low-*T*_c_ phases, suggesting that the (NH_3_)_y_Na_x_FeSe sample showing the *T*_c_ of 32 K contains NH_3_. The value is close to that, y = 0.3, for the ‘NH_3_-poor’ phase exhibiting the high *T*_c_ of 45 K in Guo’s paper. As seen from *T*_c_ and lattice constants (*a* and *c*) shown in Table 1, our low-*T*_c_ phase (*T*_c_ = 32 K) is different from the NH_3_-free phase (*T*_c_ = 37 K)^9^, while our high-*T*_c_ phase may be the same as NH_3_-poor phase (*T*_c_ = 45 K)^9^. Thus, (NH_3_)_y_Na_x_FeSe possesses at least four superconducting phases (*T*_c_ = 32.5, 37, 42 and 45–46 K).

It is significant that the occupancy of Li is fractional at x < 1.5 in (NH_3_)_y_Li_x_FeSe (off-center structure), implying that the site occupancy may change continuously in the range x = 0.1 to 0.9. Therefore, the continuous variation of *c* (Fig. 3(d)) may be due to this change of occupancy. In other words, changes in the presence and absence of Li at 4*c* and 2*b* sites may be what produce the continuous variation of *c*. In (NH_3_)_y_Li_x_FeSe, the presence of some Li at these off-center positions is maintained through the x range of 0.1 to 0.9, in contrast to the progression from on-center to off-center occupancy by Na in (NH_3_)_y_Na_x_FeSe. This difference can be attributed to the different ionic radii of Na and Li.

A recent study reported^10^ that the *T*_c_ *vs. c* plot of (NH_3_)_y_M_x_FeSe showed saturated behavior, *i.e., T*_c_ increases up to 45 K and then becomes saturated with a monotonic increase in *c*. However, the origin of this saturation behavior^10^ is different from that observed in (NH_3_)_y_Li_x_FeSe (Fig. 3(b)) because the *c* does not increase monotonically against x in (NH_3_)_y_Li_x_FeSe. Here, we must ask the question why *c* shows a maximum at x = 0.5, regardless of the simple prediction that an increase in the amount of Li would cause more expansion of FeSe plane spacing. The local structure around the Li atoms may change depending on x, *i.e.*, the local structure around M (number of NH_3_, molecular orientation…) may be different at different x’s. At the present stage, we note the possibility that the local structure best able to expand the plane-spacing is realized at x = 0.5, although no direct evidence has yet been obtained.

(NH_3_)_y_Na_x_FeSe has more than two different superconducting phases (high-*T*_c_ (or NH_3_-poor) and low-*T*_c_ phases in addition to NH_3_-free and NH_3_-rich phases^9^), while (NH_3_)_y_Li_x_FeSe has only a single phase with the high *T*_c_. In addition, we recently found that (NH_3_)_y_Cs_x_FeSe possessed only a single phase with low *T*_c_ (not shown). This is because the Cs metal atom may not occupy off-center positions: the full occupation of those off-center positions would be difficult in view of the larger ionic radius of Cs. Thus, in this paper we conclude that metal occupation is the most significant key to determine the lattice constants and *T*_c_. The influence of amount and molecular orientation of NH_3_ should be further investigated. Detailed structural analysis of (NH_3_)_y_M_x_FeSe using the Rietveld refinement of neutron diffraction results is necessary to confirm the exact structure of (NH_3_)_y_Na_x_FeSe, which would make the effect of structure (occupation sites) on *T*_c_ unequivocal.

## Methods

### Sample preparation and characterizations

The β-FeSe samples were prepared by the annealing method described in Ref. 6. The samples of (NH_3_)_y_M_x_FeSe (M: Na and Li) were synthesized using the liquid NH_3_ technique as described in Ref. 6. All the experimental procedures are the same as in our previous reports^5^,^8^.

The DC magnetic susceptibility, *M* / *H*, of all samples was measured using a SQUID magnetometer (Quantum Design MPMS2); *M* and *H* refer to magnetization and applied magnetic field, respectively. The *M* / *H* in this paper corresponds to mass magnetic susceptibility (cm^3^ g^−1^ = emu g^−1^). The XRD patterns of the samples were measured with a Rigaku R-AXIS RAPID-NR X-ray diffractometer with Mo *K*α source (wavelength λ = 0.71078 Å). The samples were introduced into quartz tubes in an Ar-filled glove box for *M* / *H* measurements, while they were introduced into capillaries for XRD. The EDX was measured with an EDX spectrometer equipped with a scanning electron microscope (SEM) (KEYENCE VE-9800 - EDAX Genesis XM_2_).

The onset superconducting transition temperature (*T*_c_^onset^) of all pristine β-FeSe samples prepared in this study was 8.5 K, and the shielding fraction at 2.0 K was ~100%. The XRD patterns of all β-FeSe samples were consistent with each other, and the lattice constants, *a* and *c*, of one β-FeSe sample were determined using LeBail refinement. The lattice constants, *a* (3.77179(4) Å) and *c* (5.5234(1) Å), of the sample were also consistent with published values^5^,^11^,^12^.

## Additional Information

**How to cite this article**: Zheng, L. *et al.* Emergence of Multiple Superconducting Phases in (NH3)_y_M_x_FeSe (M: Na and Li). *Sci. Rep.* **5**, 12774; doi: 10.1038/srep12774 (2015).

## Supplementary Material

Supplementary Information

## Figures and Tables

**Table 1:** Structure and *T*_c_ of superconducting phases found for (NH_3_)_y_Na_x_FeSe.

	**composition**	**a (Å)**	**c (Å)**	**d (Å)**	***T*_c_ (K)**
low-*T*_c_^a^ phase	(NH_3_)_y_Na_0.28(3)_Fe_0.77(4)_Se	3.889 (2)	14.257 (7)	7.12	32.5
high-*T*_c_^a^ phase	(NH_3_)_y_Na_1.1(1)_Fe_0.71(3)_Se	3.8266 (7)	17.565 (9)	8.78	46
NH_3_-free^b^	Na_0.65(1)_Fe_1.93(1)_Se_2_	3.7870 (4)	13.6678 (4)	6.83	37
NH_3_-poor^b^	(NH_3_)_0.6_Na0._80(4)_Fe_1.86(1)_Se_2_	3.7991 (2)	17.4165 (4)	8.71	45
NH_3_-rich^b^	—	—	—	11.1	42

^a^Taken from this study. The data corresponds to Fig. 2(d).

^b^Taken from ref. 9.

## References

[b1] Ying T. P. *et al.* Observation of superconductivity at 30–46K in A_x_Fe_2_Se_2_ (A=Li, Na, Ba, Sr, Ca, Yb, and Eu). Sci. Rep. 2, 426 (2012). 22645642 10.1038/srep00426 PMC3361284

[b2] Burrard-Lucas M. *et al.* Enhancement of the superconducting transition temperature of FeSe by intercalation of a molecular spacer layer. Nature Mater. 12, 15–19 (2013). 23104153 10.1038/nmat3464

[b3] Sedlmaier S. J. *et al.* Ammonia-rich high-temperature superconducting intercalates of iron selenide revealed through time-resolved *in Situ* X-ray and Neutron diffraction. J. Am. Chem. Soc. 136, 630–633 (2014). 24354523 10.1021/ja411624q

[b4] Ying T. P. *et al.* Superconducting Phases in Potassium-Intercalated Iron Selenides. J. Am. Chem. Soc. 135, 2951–2954 (2013). 23406203 10.1021/ja312705x

[b5] Zheng L. *et al.* Superconductivity in (NH_3_)_y_Cs_0.4_FeSe. Phys. Rev. B 88, 094521 (2013).

[b6] Sun L. L. *et al.* Re-emerging superconductivity at 48 kelvin in iron chalcogenides. Nature 483, 67–69 (2012). 22367543 10.1038/nature10813

[b7] Ye G. J. *et al.* Superconductivity in Yb_x_*M*_y_HfNCl (*M* = NH_3_ and THF). Phys. Rev. B 86, 134501 (2012).

[b8] Sakai Y. *et al.* Superconducting phases in (NH_3_)_y_*M*_x_FeSe_1−z_Te_z_ (*M* = Li, Na, and Ca). Phys. Rev. B 89, 144509 (2014).

[b9] Guo J. G., Lei H. C., Hayashi F. and Hosono H. Superconductivity and phase instability of NH_3_-free Na-intercalated FeSe_1-z_S_z_. Nat. Commun. 5, 4756 (2014). 25154371 10.1038/ncomms5756

[b10] Noji T. *et al.* Synthesis and post-annealing effects of alkaline-metal-ethylenediamine-intercalated superconductors A_x_(C_2_H_8_N_2_)yFe_2−z_Se_2_ (A = Li, Na) with T_c_ = 45 K. Physica C 504, 8–11 (2014).

[b11] McQueen T. M. *et al.* Extreme sensitivity of superconductivity to stoichiometry in Fe_1+δ_Se. Phys. Rev. B 79, 014522 (2009).

[b12] Hsu F.-C. *et al.* Superconductivity in the PBO-type structure a-FeSe, Proc. Natl. Acad. Sci. 105, 14262–14264 (2008). 18776050 10.1073/pnas.0807325105 PMC2531064

