# Supplementary material for: Emergence of Multiple Superconducting Phases in (NH3)yMxFeSe (M: Na and Li)
Source: Sci Rep. 2015 Aug 4;5:12774. doi: 10.1038/srep12774 (PMC4536530; doi:10.1038/srep12774)
Supplement: Supplementary Information [file srep12774-s1.pdf]

Supplemental Information

Emergence of Multiple Superconducting Phases in $(NH_3)_yM_xFeSe$ (M: Na and Li)

Lu Zheng[1], Xiao Miao[1], Yusuke Sakai[1], Masanari Izumi[1], Hidenori Goto[1], Saki Nishiyama[1], Eri Uesugi[1], Yuichi Kasahara[2], Yoshihiro Iwasa[2] and Yoshihiro Kubozono[1,3,*]

[1] Research Laboratory for Surface Science, Okayama University, Okayama 700-8530, Japan

[2] Department of Applied physics, The University of Tokyo, Tokyo 113-8564, Japan

[3] Research Center of New Functional Materials for Energy Production, Storage and Transport, Okayama University, Okayama 700-8530, Japan

Contents of Supplementary Information

1) EDX spectrum of the low-T_c phase in $(NH_3)_yNa_xFeSe$ (nominal x = 0.1)
2) EDX spectrum of the high-T_c phase in $(NH_3)_yNa_xFeSe$ (nominal x = 1.0).
3) Plot of *a vs*. x in $(NH_3)_yNa_xFeSe$; x was determined from EDX.
4) XRD pattern of $(NH_3)_yLi_xFeSe$ (nominal x = 0.1) together with the pattern calculated by LeBail fitting.
5) x dependence of *a* in $(NH_3)_yLi_xFeSe$; x is nominal value.

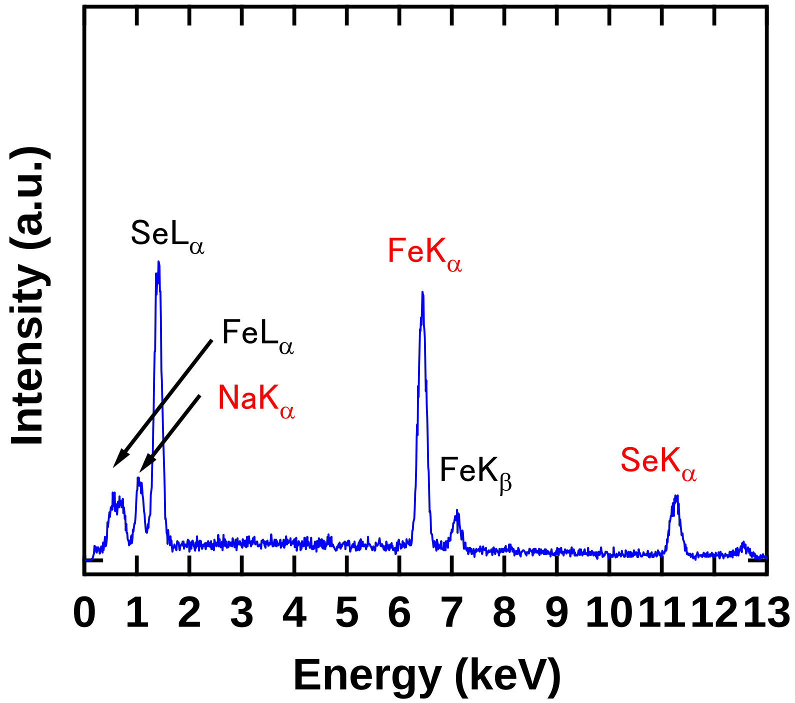

Figure S1. EDX spectrum of the low-T_c phase in $(NH_3)_yNa_xFeSe$ (nominal x = 0.1). The peaks shown by red characters are used for a determination of stoichiometric composition.

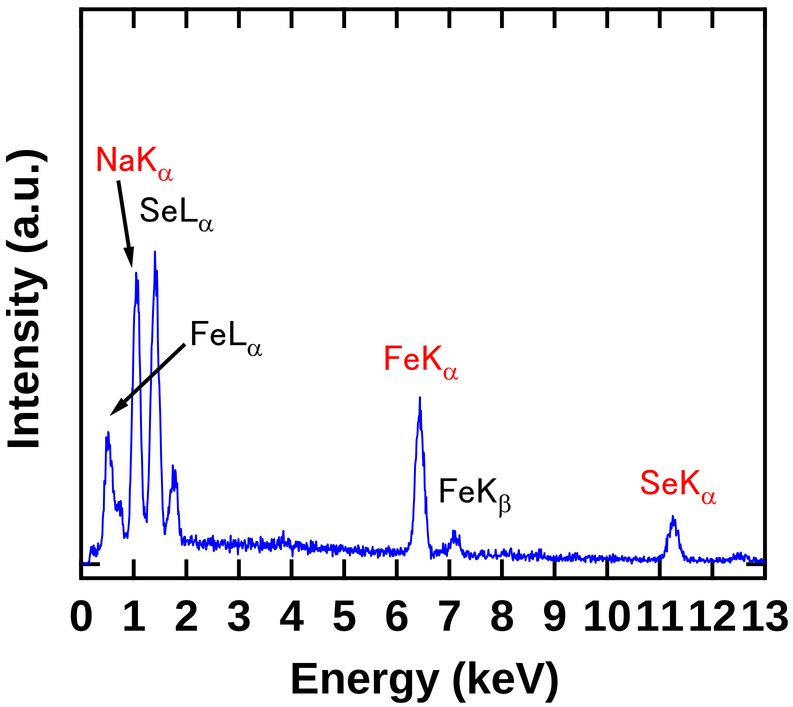

Figure S2. EDX spectrum of the high-T_c phase in $(NH_3)_yNa_xFeSe$ (nominal x = 1.0). The peaks shown by red characters are used for a determination of stoichiometric composition.

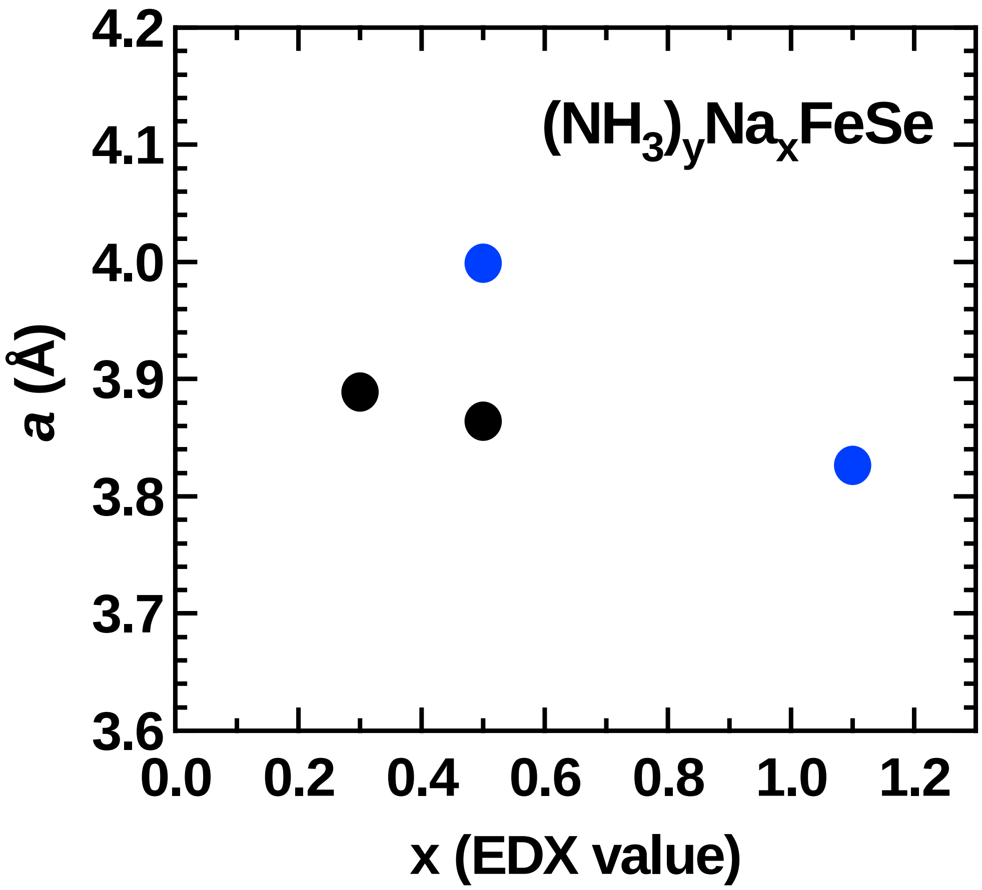

Figure S3. Plot of *a vs*. x in $(NH_3)_yNa_xFeSe$; x was determined from EDX.

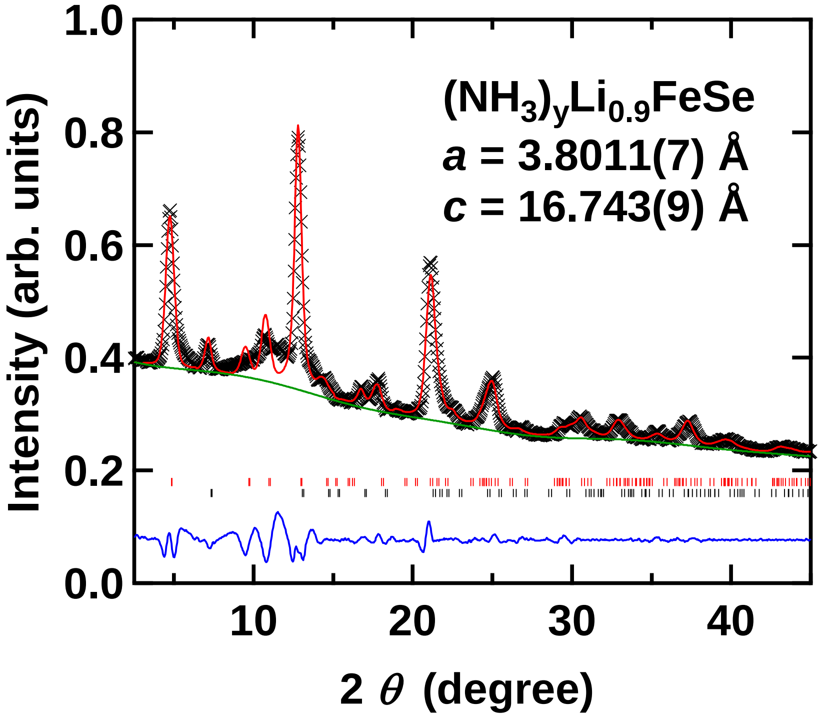

Figure S4. XRD pattern of $(NH_3)_yLi_xFeSe$ (nominal x = 0.1) together with the pattern calculated by LeBail fitting.

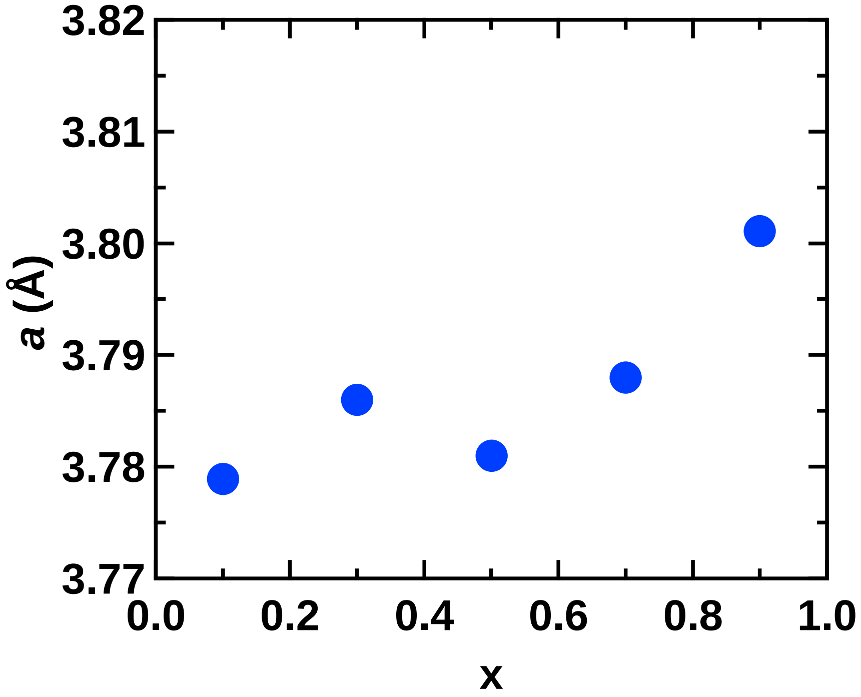

Figure S5. x dependence of *a* in $(NH_3)_yLi_xFeSe$; x is nominal value.
